# A Study of Hypertension and Fitness of Individuals with Spinal Cord Injury: A Cross-Sectional Study

**DOI:** 10.3390/healthcare12212114

**Published:** 2024-10-23

**Authors:** Bogja Jeoung, Sunghae Park

**Affiliations:** 1 Department of Exercise Rehabilitation, Gachon University, Incheon 21936, Republic of Korea; bogja05@gachon.ac.kr; 2 Department of Exercise Rehabilitation, Gachon University, Seongnam 13120, Republic of Korea

**Keywords:** physical function, disabilities, blood pressure, body composition

## Abstract

**Background/Objectives:** Individuals with spinal cord injuries have a higher incidence of chronic conditions such as hypertension and cardiovascular diseases due to a sedentary lifestyle and low levels of physical activity caused by their disability. Additionally, their physical fitness levels are lower compared to those without disabilities. This cross-sectional study aimed to investigate the relationship between hypertension and the fitness of individuals with spinal cord injuries in South Korea while considering differences across sexes and spinal cord injury levels. **Methods:** This study used data for 835 individuals with spinal cord injuries aged 20–64 years who visited the Korea Paralympic Committee fitness standard test centers from 2018 to 2022, obtained from the Korea Culture Information Sports Association’s big data market. The data were analyzed using a series of *t*-tests, a one-way analysis of variance, a logistic regression analysis, and the four-quartile method. **Results:** The prevalence of hypertension was 24.4%, and it was different according to the spinal cord injury impairment level. A lower grip strength, a lower arm curl, and a higher body mass index were associated with increased blood pressure. **Conclusions:** Therefore, a high level of physical strength in people with spinal cord injuries is thought to contribute to lowering blood pressure.

## 1. Introduction

The term “vulnerable groups” generally refers to populations that, in comparison to broader society, face significant barriers to social participation. These barriers often arise from economic, physical, and other conditions that limit their opportunities and increase the likelihood of their exclusion from equal participation in societal benefits. Among these groups, individuals with disabilities are particularly disadvantaged, as they experience notably reduced access to healthcare services and encounter higher risk factors [1,2]. Consequently, this population necessitates targeted intervention at the national level to ensure equitable healthcare access and to address their specific needs.

A spinal cord injury (SCI) is defined as damage to the spinal cord, a part of the central nervous system, caused by a disease or accident that results in the complete or partial paralysis of the trunk and limbs, as well as sensory and motor function limitations [3,4]. Spinal nerve damage can lead to muscle paralysis, partial paralysis, and spasms in the trunk, arms, and legs, depending on the location and extent of the injury. Therefore, individuals with an SCI experience complex problems, such as muscle spasms, bladder and digestive issues, urinary infections, intestinal problems, osteoporosis, bedsores, and obesity [3,4]. People with spinal cord disorders rely on wheelchairs for most of their daily life; thus, their movement is limited. Additionally, their long sedentary life negatively affects their blood pressure and heart rate at rest. Hence, SCIs are a major cause of death because the prevalence of chronic diseases such as high blood pressure, cerebrovascular disease, and diabetes in patients with an SCI are 3.4, 4, and 1.4 times higher, respectively, than those of people without a disability [4,5].

Individuals with an SCI present with various functional impairments, depending on the SCI level. A cervical cord injury can impair thermoregulation and upper-limb function, resulting in reduced physical activity. Furthermore, when an SCI occurs above the sixth thoracic vertebra, it can lead to severe respiratory impairment during exercise, resulting in respiratory difficulties. Individuals with SCIs above the sixth thoracic vertebra have been shown to have a lower muscular strength and endurance than those with injuries below the sixth thoracic vertebra [6,7,8]. Cardiovascular endurance and muscular strength are essential health-related components of fitness. Individuals with an SCI reportedly exhibit an approximately 30% reduction in cardiovascular endurance compared with people without disabilities with similar demographic conditions. Additionally, their muscular strength is reportedly impaired by 30–50% [9,10]. Owing to their disability, more than half of the individuals with SCIs experience the negative effects of a sedentary lifestyle and low physical activity on body composition, such as being overweight or obese. Obesity (especially visceral fat) is particularly prevalent around the abdominal area in individuals with an SCI [10]. Increased visceral fat is an important risk factor for an increased prevalence of diabetes, metabolic syndrome, and hypertension [11,12,13].

Individuals with SCIs exhibit significant differences in fitness levels according to their type of participation in physical activity and their use of wheelchairs owing to their level of injury. This can contribute to variations in the occurrence rates of secondary complications, including cardiovascular diseases, metabolic disorders, muscle atrophy, and musculoskeletal disorders [14]. The effective management of fitness factors in individuals with SCIs can prevent secondary complications and improve exercise function [15]. Thus, improving and maintaining physical fitness are essential for enhancing the quality of life of people with disabilities.

Since the 1980s, numerous researchers have attempted to understand the relationship between fitness and health. Currently, maintaining a certain level of cardiovascular fitness is now known to diminish the risk of various health conditions such as hypertension, obesity, diabetes, coronary artery disease, several types of cancer, and other health disorders [8,16]. Previous studies investigating the relationship between fitness and hypertension in people both with and without disabilities have shown that cardiovascular fitness, muscular strength, muscular endurance, and obesity play roles in increasing blood pressure [17,18,19]. As suggested by many previous studies, improvements in fitness resulting from participation in physical activities lead to various health benefits. A high level of health-related physical fitness is a strong, well-known predictor of a reduced prevalence of and reduced mortality rates from metabolic syndrome, type 2 diabetes, and cardiovascular diseases [20,21]. A previous study, although on non-disabled people, evaluated the relative risk of cardiovascular diseases in relation to physical activity and fitness and revealed that this risk decreased by approximately 25% with an increase in physical activity and decreased by approximately 60% with an increase in fitness levels. Interestingly, physical activity gradually declined, whereas the fitness levels showed a 40% drastic reduction in the relative risk of cardiovascular diseases, even when the fitness levels reached the bottom 25%, suggesting that fitness has greater health-related value than physical activity [22]. Vivodtzew et al. [23] and Sisto et al. [24] suggested that improving all components of physical fitness, including cardiorespiratory endurance, strength and muscular endurance, flexibility, and body composition, may alleviate secondary health complications associated with a chronic SCI and enhance functional capacity, thus positively impacting the overall health in individuals with SCIs. These studies provide evidence that promoting exercise and enhancing physical fitness can positively affect the overall health of individuals with an SCI and reduce the risk of secondary health complications associated with chronic conditions. However, they did not examine the relative risk of cardiovascular disease associated with the fitness levels of people with an SCI. To address this gap and confirm this notion, this study aimed to investigate the relationship between the physical fitness of people with SCIs and their blood pressure.

## 2. Materials and Methods

### 2.1. Data Collection

The present study adopted a cross-sectional design. The data were obtained from the Korea Culture Information Sports Association’s big data market (https://www.bigdata-culture.kr/bigdata/user/data_market/agency/detail.do?id=kspo_org, accessed on 28 November 2023), which is publicly available. At the time of data collection, 12 Korea Paralympic Committee (KPC) fitness standard test centers released data covering 5 years (2018–2022). All the data were used except for data errors; this study used data of 835 (male: 545, 65.3%; female: 290, 34.7%) people with SCIs, aged 20–64 years (mean age: 46.5 ± 12.9), who voluntarily visited the centers.

The participants were classified according to the spinal injury area as follows: T6 over (*n* = 146; 17.5%), T6 under (*n* = 593; 71%), and quadriplegia (*n* = 96; 11.5%).

### 2.2. Health-Related Physical Fitness and Blood Pressure Test

The health-related physical fitness tests designed for individuals with SCIs evaluate five components: (i) cardiovascular endurance, using a 5 min wheelchair run test; (ii) upper-body muscle strength, assessed through hand grip strength; (iii) muscle endurance, measured with a 2 min dumbbell bicep curl test; (iv) flexibility, assessed via the shoulder back scratch test; and (v) body composition, using BMI and body fat percentage [25,26,27,28]. In this research, all the components of physical fitness (i–iv) and body composition (v) were evaluated at 12 KPC fitness standard test centers for individuals with disabilities. Each of these fitness tests demonstrated a strong internal consistency, with Cronbach’s alpha reliability values ranging from α = 0.70 to α = 0.93 [25,26,27,28]. Certified instructors with national qualifications in health and fitness conducted the assessments.

First, cardiovascular endurance was assessed through the 5 min wheelchair run test. In this test, participants completed a 20 m shuttle run in a wheelchair for a duration of 5 min. The total distance they traveled was calculated by counting the number of full round trips, and any partial distances were measured using a ruler marked in 1 m increments along the 20 m course.

Second, upper-body muscle strength was assessed using the handgrip strength test. The participants completed the test twice for both the left and right hands while seated in their wheelchairs, using a digital hand dynamometer (TKK 5401, Osaka, Japan). The highest measurement was recorded to the nearest 0.1 kg.

Third, upper-body muscle endurance was measured using the dumbbell bicep arm curl 2 min test. The participants performed this test twice for the left- and right-arm curls, with a dumbbell weight of 4 kg for males and 2 kg for females. The participants were instructed to curl toward the chest, return straight, and continue to perform as many repetitions as possible within 2 min. The highest number of repetitions was recorded between the left and right arms.

Fourth, shoulder flexibility was assessed using the shoulder back scratch test. The participants were instructed to place their right hand over their head, reach it behind their back, place the other hand behind their waist/back, and try to touch the fingers of both middle fingers behind their back. Conversely, the left hand was raised above the head and sent behind the back, and the other hand was sent behind the waist/back so that the fingers of both middle fingers touched. Each participant performed the action twice, and measurements were recorded to the nearest score, with a recording unit of 0.1 cm. Finally, regarding body composition, BMI was calculated by dividing the body weight in kilograms (kg) by the height in meters squared (m^2^). The body fat percentage was measured through a bioelectrical impedance analysis using an InBody S10 device (InBody Co., Seoul, Republic of Korea). The systolic blood pressure (SBP) and diastolic blood pressure (DBP) were measured using an automatic blood pressure device (BPBIO 320S; Inbody Co., Seoul, Republic of Korea) after the chair was stabilized for approximately 10 min. According to the 2018 Korean Society of Hypertension Guidelines, the standard for hypertension is defined as normal for <120 mmHg, elevated blood pressure for 120–129, prehypertension for 130–139 mmHg, and hypertension for >140 mmHg.

### 2.3. Data Analysis

The data were analyzed using SPSS 26.0 (IBM Corp., Armonk, NY, USA). An exploratory data analysis was conducted to assess the normality of the data. Descriptive statistics were calculated to determine the means, standard deviations, frequencies, and percentages of the variables. For the main analysis, paired *t*-tests were initially used to examine the differences in body composition and physical fitness scores between males and females, followed by a one-way analysis of variance (ANOVA) to compare physical fitness across different blood pressure levels.

If a significant difference was found, Scheffé’s post hoc test was used to identify which specific pairs of means were significantly different. Before this, the Mahalanobis distance was calculated to check for multivariate outliers, using a critical chi-squared value at *p* = 0.05, and any identified outliers were removed from the dataset. A multiple logistic regression analysis, adjusted for both sex and age, was performed to explore the relationship between the participants’ physical fitness factors and blood pressure. The significance threshold for all the tests was set at *p* < 0.05.

In this study, *t*-tests were employed to compare the physical fitness scores between males and females due to the suitability of this test for analyzing differences between two independent groups. A one-way ANOVA was used to compare the physical fitness across different blood pressure levels, as it allows for the comparison of more than two groups, providing robustness in analyzing multiple categories. A logistic regression analysis was applied to investigate the relationship between physical fitness factors and blood pressure while adjusting for potential confounders such as age and sex. Logistic regression is appropriate for modeling binary outcomes like hypertension. All the statistical tests were chosen based on the distribution and nature of the data, with assumptions like normality verified through an exploratory data analysis.

## 3. Results

Table 1 summarizes the detailed characteristics of the participants. The participants’ ages ranged from 20 to 64 years (age mean: 46.5 ± 12.9). Among the participants, 65.3% were males, whereas 34.7% were females. The BMI, arm curl, and 5 min wheelchair shuttle run did not differ between males and females. However, in the fitness tests, the values for grip strength were higher in males than in females. Furthermore, females exhibited a better performance in the shoulder back scratch test and showed a higher body fat percentage than males. Blood pressure (SBP and DBP) was higher in males than in females.

Table 2 shows the differences in the prevalence of hypertension according to sex and disability level. The prevalence rates of hypertension were 28.7% in males with SCIs and 16.2% in females with SCIs. According to the disability levels, the highest and lowest prevalence of hypertension in males was under the “T6 over” and “quadriplegia” classifications, respectively, whereas the highest and lowest prevalence of hypertension in females was under the “quadriplegia” and “T6 over” classifications, respectively.

Table 3 shows the differences in fitness according to the blood pressure stage. The results indicated differences in the grip strength, arm curl, and BMI according to the blood pressure stage. The grip strength showed a significant difference according to blood pressure (post hoc, normal > elevated blood pressure, prehypertension, hypertension; *p* < 0.001). Similarly, the arm curl and BMI also showed a significant difference according to blood pressure (for arm curl: post hoc, elevated > prehypertension, hypertension, *p* < 0.05; for BMI: post hoc, normal > prehypertension, hypertension, *p* < 0.001).

Table 4 shows the differences in fitness according to age. The results indicated differences in the grip strength of males, the arm curl of all, and the BMI of all and males (*p* < 0.05). In terms of the grip strength, men in their 30s demonstrated the best performance, while those in their 20s showed the lowest. For women, the grip strength was highest in their 40s and lowest in their 30s. In tests measuring aerobic capacity, such as the 5 min wheelchair run, and flexibility, such as the shoulder back scratch, no significant differences were observed across age groups. Regarding body fat percentage, as measured by BMI, men in their 30s had the highest values, whereas those in their 20s had the lowest. In women, BMI was lowest in their 30s and highest in their 40s.

The risks of elevated blood pressure, prehypertension, and hypertension were confirmed on the basis of the odds ratios for blood pressure stage and fitness level and are displayed in Table 5. The grip strength increased the risk of hypertension by 2.02 times in level 2 compared with level 1, 2.126 times in level 3 compared with level 1, and 4.98 times in level 4 compared with level 1. The muscular endurance, as measured using the arm curl test, increased the risk of elevated blood pressure and hypertension by 0.48 times in level 3 compared with level 1 and by 2.52 times in level 4 compared with level 1, respectively. Finally, the BMI increased the risk of elevated blood pressure, prehypertension, and hypertension by 0.259 times in the obese group compared with the normal group. However, there was no significant difference in levels 2 and 3 compared with level 1 for elevated blood pressure. Furthermore, there were no significant differences between levels 1 and 2 for prehypertension and hypertension across all fitness levels.

## 4. Discussion

Many studies are attempting diverse research with an interest in exercise and fitness levels to prevent the onset of chronic disease. It has been reported that individuals with higher levels of physical fitness have a lower risk of developing hypertension. Among the components of physical fitness, those with higher performance levels in cardiovascular endurance, muscular strength, and muscular endurance have been reported to contribute to a reduced incidence of hypertension compared to those with lower levels [17,18,19,21,29]. This study attempted to figure out the relationship between physical strength and blood pressure according to the degree of spinal cord injury in people with spinal cord injuries, who have vulnerable health. There was a difference between males and females in terms of the prevalence of hypertension based on the SCI level. Specifically, males with an SCI at the T6 level or above and females with quadriplegia had a higher prevalence of hypertension than their counterparts. Jeoung [30] suggested a relationship between SCI levels and unstable blood pressure. It was especially reported that individuals with an SCI at the T6 level or above exhibit more unstable blood pressure than those with an SCI below T6. This is because a reduction in the stimulation of sympathetic nervous system neurons leads to an impairment in the secretion of catecholamines from the adrenal glands, affecting cardiovascular function and myocardial contractions [31,32]. Such unstable blood pressure is associated with negative outcomes, including cerebrovascular disease, vascular cognitive impairment, and an increased risk of stroke. Among the fitness factors of individuals with SCIs, muscle strength and endurance test items, such as the hand grip strength and arm curl, influence hypertension. In a cohort study by Taekema et al. [33] that investigated the relationship between handgrip strength and hypertension, no significant correlation was found among middle-aged participants. However, in studies targeting individuals aged ≥85 years, a strong association between hand grip strength and hypertension was found. Ji et al. [34] investigated the relationship between hand grip strength and hypertension among people without disabilities and found a strong correlation among males, whereas females showed no significant correlation. Jeoung et al. [35] reported a strong correlation between blood pressure, muscle strength, and endurance in middle-aged individuals. As shown in these previous studies, the findings indicate a strong correlation between blood pressure and handgrip strength; however, these studies present conflicting results. Feng et al. [36] reported that higher hand grip strength scores are associated with a lower risk of hypertension [37,38,39,40]. These studies were conducted on the association between hypertension and strength in people without disabilities; only a few studies have been conducted on people with SCIs. This study is significant, as it is the first to reveal the relationship between hypertension and muscle strength, as well as the differences in the prevalence of hypertension according to the level of spinal cord injury. In future research, it will be necessary to elucidate the mechanism underlying the association between hand grip strength and hypertension and clarify how much hypertension can be lowered according to the level of grip strength intensity.

## 5. Limitations and Strength of This Study

This study faced certain limitations, primarily related to the lack of consistency in the measurement methods, which arose due to its cross-sectional design and reliance on open-source data for assessing fitness indicators. Additionally, the study was unable to account for comorbidities or exercise history among the participants. Despite these limitations, the study holds significant value as the first to explore the relationship between blood pressure and physical fitness in individuals with spinal cord injuries (SCIs). The findings of this research have important implications for policymakers, offering valuable insights for monitoring the physical health of individuals with SCIs and developing tailored activity and exercise programs. Regular participation in sports and exercise may contribute to healthier lifestyles for individuals with SCIs, promoting an improved overall well-being in the future.

## 6. Conclusions

This study investigated the relationship between blood pressure and physical fitness in adults with SCIs aged 20–64 years using data from the KPC fitness standard test centers, which released data covering 5 years (2018–2022). Significant differences in the body fat percentage, grip strength, arm curl, shoulder back scratch, SBP, and DBP were observed between the sexes. In addition, the prevalence of hypertension differed according to the SCI level. It has been confirmed that an increase in fitness levels contributes to a reduction in blood pressure. Specifically, a low grip strength, a reduced arm curl, and a high BMI contribute to raising blood pressure.

In this study, we utilized the STROBE statement—checklist of items that should be included in reports of cross-sectional studies, which can be found in the Appendix A.

## Figures and Tables

**Table 1:** Physical fitness of individuals with SCIs (*n* = 835).

	Total (*n* = 835)		Males (*n* = 545) 65.3%	Females (*n* = 290) 34.7%	*p*
Age (20–64 yr)	46.5 ± 12.9		45.91 ± 13.2	47.66 ± 12.2	<0.063
Disability level	T6 over	146 (17.5%)	106 (19.4%)	40 (13.8%)	
	T6 under	593 (71.0%)	381 (69.9)	212 (73.1%)	
	Quadriplegia	96 (11.5%)	58 (10.6%)	38 (13.1%)	
Height (cm)	162.0 ± 11.09		166.5 ± 9.0	153.5 ± 9.4	<0.001 ***
Weight (kg)	63.8 ± 14.9		67.9 ± 14.9	56.1 ± 11.5	<0.001 ***
Body mass index (kg/m^2^)	24.2 ± 4.6		24.4 ± 4.6	23.8 ± 4.8	<0.055
Body fat percentage (%)	33.1 ± 11.5		30.7 ± 11.5	37.9 ± 10.2	<0.001 ***
Grip strength (kg)	28.8 ± 12.8		32.9 ± 13.0	21.2 ± 8.1	<0.001 ***
Arm curl (times)	87.4 ± 40		87.4 ± 40	87.3 ± 41.8	<0.493
5 min wheelchair run (m)	369.7 ± 174.0		378.2 ± 176.3	346.8 ± 167.3	<0.146
Shoulder back scratch (cm)	−16.8 ± 16.8		−19.0 ± 17.3	−12.2 ± 14.7	<0.001 ***
SBP (mmHg)	128.0 ± 16.1		129.8 ± 156.5	124.7 ± 14.8	<0.001 ***
DBP (mmHg)	81.0 ± 13.1		82.6 ± 12.8	78.0 ± 13.0	<0.001 ***

*** *p* < 0.001; SCI, spinal cord injury; SBP, systolic blood pressure; DBP, diastolic blood pressure.

**Table 2:** Prevalence of hypertension in participants with SCIs.

	Classification	Normal, <120 mmHg (a)	Elevated Blood Pressure, 120–129 mmHg (b)	Prehypertension, 130–139 mmHg (c)	Hypertension, ≥140 mmHg (d)
	Total	210 (25.2%)	255 (30.6%)	165 (19.8%)	203 (24.4%)
	Male	121 (22.3%)	152 (28%)	114 (21%)	156 (28.7%)
	Female	89 (30.7%)	103 (35.5%)	51 (17.6%)	47 (16.2%)
Total	T6 over	43 (29.7%)	41 (28.3%)	22 (15.2%)	39 (26.9%)
	T6 under	135 (22.8%)	188 (31.7%)	130 (21.9%)	140 (23.6%)
	Quadriplegia	32 (33.7%)	26 (27.4%)	13 (13.5)	24 (25.3%)
Male	T6 over	28 (26.7%)	24 (22.6%)	17 (16.2%)	36 (34.3%)
	T6 under	73 (19.2%)	112 (29.4%)	89 (23.4%)	107 (28.1%)
	Quadriplegia	20 (35.1%)	16 (28.1%)	8 (14%)	13 (22.8%)
Female	T6 over	15 (37.5%)	17 (42.5%)	5 (12.5%)	3 (7.5%)
	T6 under	62 (29.2%)	76 (35.8%)	41 (19.3%)	33 (15.6%)
	Quadriplegia	12 (31.6%)	10 (26.3%)	5 (13.2)	11 (28.9%)

SCI, spinal cord injury.

**Table 3:** Differences in physical fitness according to hypertension among people with SCIs.

	Normal, <120 mmHg (a)	Elevated Blood Pressure, 120–129 mmHg (b)	Prehypertension, 130–139 mmHg (c)	Hypertension, ≥140 mmHg (d)	*F*	*p*	Post Hoc
Grip strength (kg)	31.39 ± 12.0	30.76 ± 12.8	29.6 ± 12.8	23.6 ± 12.2	32.116	<0.001 ***	b, c, d < a
Arm curl (times)	87.2 ± 36.3	97.1 ± 41.0	91.8 ± 41.4	79.6 ± 39.2	5.12	<0.024 *	c, d < b
5 min wheelchair run (m)	399.89 ± 159.4	397.49 ± 178.5	379.9 ± 171.0	300.62 ± 179.5	6.43	<0.076	-
Shoulder back scratch (cm)	−17.4 ± 16.1	−14.8 ± 16.2	−18.5 ± 18.0	16.9 ± 17.0	0.144	<0.341	-
BMI (kg/m^2^)	22.7 ± 4.4	24.09 ± 4.3	24.9 ± 4.5	25.4 ± 4.9	38.83	<0.001 ***	c, d < a

*** *p* < 0.001, * *p* < 0.05; tested by performing one-way analysis of variance. Post hoc, Scheffé; Grip strength, arm curl, BMI (body mass index).

**Table 4:** Differences in physical fitness according to age.

		20–29 (a)	30–39 (b)	40–49 (c)	50–59 (d)	60–64 (e)	*p*	Post Hoc
Grip strength (kg)	Total	26.2 ± 15.1	31.2 ± 16.3	29.7 ± 12.7	29.5 ± 11.3	27.6 ± 10.6	0.05 *	-
	Male	29.5 ± 15.5	37.7 ± 15.3	32.2 ± 113.6	34.6 ± 10.8	30.7 ± 10.6	0.001 ***	a > b, b > e
	Female	18.6 ± 11.3	18.5 ± 9.3	23.9 ± 7.9	22.0 ± 7.0	20.4 ± 6.3	0.01 **	-
Arm curl (times)	Total	76.2 ± 44.3	92.8 ± 42.4	89.8 ± 39.3	94.1 ± 39.4	78.7 ± 33.8	0.01 *	a > d
	Male	78.6 ± 46.4	95.1 ± 40.0	87.2 ± 38.2	95.2 ± 36.6	78.2 ± 36.6	0.05 *	-
	Female	69.3 ± 38.3	87.2 ± 48.3	96.6 ± 41.4	92.2 ± 43.5	79.6 ± 27.8	0.12	-
5 min wheelchair run (m)	Total	327.7 ± 178.5	383.7 ± 199	400.3 ± 169	367.2 ± 157	354.3 ± 177	0.41	-
	Male	347.2 ± 178.4	406.2 ± 204.5	389.4 ± 173.3	380.5 ± 160.5	348.4 ± 201.2	0.88	-
	Female	291.5 ± 180.0	333.7 ± 188.7	429.6 ± 160.5	311.2 ± 136.4	374.1 ± 76.5	0.27	-
Shoulder back scratch (cm)	Total	−18.8 ± 19.9	16.4 ± 15.7	−16.7 ± 19.0	−4.6 ± 15.3	−20.6 ± 13.5	0.08	-
	Male	−21.0 ± 19.8	−19.5 ± 15.3	−17.5 ± 20.2	−17.3 ± 15.4	−23.0 ± 14.0	0.32	-
	Female	−12.2 ± 19.5	−8.3 ± 14.0	−13.6 ± 15.0	−10.1 ± 14.0	−17.4 ± 12.3	0.13	-
BMI (kg/m^2^)	Total	22.4 ± 5.3	24.7 ± 5.4	24.0 ± 4.9	24.7 ± 4.0	24.8 ± 3.9	0.001 ***	a < b, d, e
	Male	22.2 ± 5.3	26.3 ± 4.9	23.8 ± 4.2	24.8 ± 3.8	25.4 ± 4.1	0.001 ***	a > b, d, e; b > c
	Female	23.1 ± 5.3	21.8 ± 5.3	24.5 ± 6.1	24.4 ± 4.2	23.6 ± 3.0	0.05 *	-

*** *p* < 0.001, ** *p* < 0.01, * *p* < 0.05; tested by performing one-way analysis of variance. Post hoc, Scheffé; Grip strength, arm curl, BMI (body mass index).

**Table 5:** Odds ratios (95% confidence intervals) for blood pressure and fitness levels compared with baseline levels.

Fitness Factor		Normal, <120 mmHg	Elevated Blood Pressure, 120–129 mmHg	Prehypertension, 130–139 mmHg	Hypertension, ≥140 mmHg
			Odds Ratio (95% Confidence Interval) with *p*-Values		
Grip strength	1st	Reference			
	2nd		1.01 (0.56–1.82) < 0.965	1.43 (0.83–2.44) < 0.19	2.02 (1.07–3.82) < 0.029 *
	3rd		1.04 (0.58–1.86) < 0.892	1.29 (0.75–2.21) < 0.357	2.12 (1.13–3.99) < 0.019 *
	4th		1.26 (0.67–2.37) < 0.460	1.68 (0.94–2.99) < 0.078	4.98 (2.65–9.35) < 0.001 ***
Arm curl	1st	Reference			
	2nd		0.75 (0.38–1.46) < 0.403	1.11 (0.59–2.10) < 0.732	0.97 (0.45–2.06) < 0.940
	3rd		0.48 (0.24–9.57) < 0.037 *	0.60 (0.31–1.16) < 0.135	1.54 (0.77–3.06) < 0.214
	4th		0.76 (0.383–1.52) < 0.448	0.93 (0.47–1.83) < 0.845	2.52 (1.25–5.09) < 0.010 **
BMI	Normal	Reference			
	Underweight		0.623 (0.263–1.478) < 0.283	0.360 (0.153–0.845) < 0.019	0.376 (0.167–0.847) < 0.018 *
	Overweight		0.578 (0.253–1.32) < 0.193	0.241 (0.106–0.546) < 0.001 ***	0.177 (0.080–0.390) < 0.001 ***
	Obesity		0.259 (0.101–0.662) < 0.005 **	0.126 (0.048–0.334) < 0.001 ***	0.109 (0.043–0.276) < 0.001 ***

*** *p* < 0.001, ** *p* < 0.01, * *p* < 0.05. BMI, body mass index.

## Data Availability

Data are contained within the article.

## Appendix A

**Table A1:** STROBE statement—checklist of items that should be included in reports of cross-sectional studies.

	Item No.	Recommendation	
Title and abstract	1	(a) Indicate the study’s design with a commonly used term in the title or the abstract	0
		(b) Provide in the abstract an informative and balanced summary of what was done and what was found	0
Introduction			0
Background/rationale	2	Explain the scientific background and rationale for the investigation being reported	0
Objectives	3	State specific objectives, including any prespecified hypotheses	0
Methods			0
Study design	4	Present key elements of study design early in the paper	0
Setting	5	Describe the setting, locations, and relevant dates, including periods of recruitment, exposure, follow-up, and data collection	0
Participants	6	(a) Give the eligibility criteria, and the sources and methods of selection of participants	0
Variables	7	Clearly define all outcomes, exposures, predictors, potential confounders, and effect modifiers. Give diagnostic criteria, if applicable	0
Data sources/measurement	8 *	For each variable of interest, give sources of data and details of methods of assessment (measurement). Describe comparability of assessment methods if there is more than one group	0
Bias	9	Describe any efforts to address potential sources of bias	0
Study size	10	Explain how the study size was arrived at	0
Quantitative variables	11	Explain how quantitative variables were handled in the analyses. If applicable, describe which groupings were chosen and why	0
Statistical methods	12	(a) Describe all statistical methods, including those used to control for confounding	0
		(b) Describe any methods used to examine subgroups and interactions	0
		(c) Explain how missing data were addressed	0
		(d) If applicable, describe analytical methods taking account of sampling strategy	0
		(e) Describe any sensitivity analyses	0
Results			0
Participants	13 *	(a) Report numbers of individuals at each stage of study—e.g., numbers potentially eligible, examined for eligibility, confirmed eligible, included in the study, completing follow-up, and analysed	0
		(b) Give reasons for non-participation at each stage	0
		(c) Consider use of a flow diagram	0
Descriptive data	14 *	(a) Give characteristics of study participants (e.g., demographic, clinical, social) and information on exposures and potential confounders	0
		(b) Indicate number of participants with missing data for each variable of interest	0
Outcome data	15 *	Report numbers of outcome events or summary measures	0
Main results	16	(a) Give unadjusted estimates and, if applicable, confounder-adjusted estimates and their precision (e.g., 95% confidence interval). Make clear which confounders were adjusted for and why they were included	0
		(b) Report category boundaries when continuous variables were categorized	0
		(c) If relevant, consider translating estimates of relative risk into absolute risk for a meaningful time period	0
Other analyses	17	Report other analyses done—e.g., analyses of subgroups and interactions, and sensitivity analyses	0
Discussion			0
Key results	18	Summarize key results with reference to study objectives	0
Limitations	19	Discuss limitations of the study, taking into account sources of potential bias or imprecision. Discuss both direction and magnitude of any potential bias	0
Interpretation	20	Give a cautious overall interpretation of results considering objectives, limitations, multiplicity of analyses, results from similar studies, and other relevant evidence	0
Generalizability	21	Discuss the generalizability (external validity) of the study results	0
Other information			0
Funding	22	Give the source of funding and the role of the funders for the present study and, if applicable, for the original study on which the present article is based	0

* Give information separately for exposed and unexposed groups. Note: An Explanation and Elaboration article discusses each checklist item and gives methodological background and published examples of transparent reporting. The STROBE checklist is best used in conjunction with this article (freely available on the Web sites of PLoS Medicine at http://www.plosmedicine.org/, Annals of Internal Medicine at http://www.annals.org/, and Epidemiology at http://www.epidem.com/). Information on the STROBE Initiative is available at www.strobe-statement.org accessed on 16 July 2024.
